# Machine learning for flow-informed aerodynamic control in turbulent wind conditions

**DOI:** 10.1038/s44172-022-00046-z

**Published:** 2022-12-16

**Authors:** Peter I. Renn, Morteza Gharib

**Affiliations:** Graduate Aerospace Laboratories, California Institute of Technology, Pasadena, CA USA

**Keywords:** Aerospace engineering, Computer science

## Abstract

Control of aerodynamic forces in gusty, turbulent conditions is critical for the safety and performance of technologies such as unmanned aerial vehicles and wind turbines. The presence and severity of extreme flow conditions are difficult to predict, and explicit modeling of fluid dynamics for control is not feasible in real time. Model-free reinforcement learning methods present an end-to-end control solution for nonlinear systems as they require no prior knowledge, can easily integrate different types of measurements, and can adapt to varying conditions through interaction. Here, we show that reinforcement learning methods can achieve effective aerodynamic control in a highly turbulent environment. Algorithms are trained with different neural network structures, and we find that reinforcement learning agents with recurrent neural networks can effectively learn the nonlinear dynamics involved in turbulent flows and strongly outperform conventional linear control techniques. We also find that augmenting state observations with measurements from a set of bioinspired flow sensors can improve learning stability and control performance in aerodynamic systems. These results can serve to inform future gust mitigation systems for unmanned aerial vehicles and wind turbines, enabling operation in previously prohibitively dangerous conditions.

## Abstract (web summary)

Renn and Gharib experimentally investigate the application of reinforcement learning to provide integrated flow information for aerodynamic control of a wing system in a highly turbulent environment. The results can inform future gust mitigation systems for unmanned aerial vehicles and wind turbines.

## Introduction

Atmospheric winds are often turbulent, containing transient flow disturbances which result in intermittent aerodynamic forces^1^. These forces affect many systems and structures, but are especially impactful on inherently aerodynamic technologies. For example, unmanned aerial vehicles (UAVs) and wind turbines both rely on fluid interaction for normal operation, but can be damaged or destroyed when operating in turbulent wind conditions^2–4^. Mitigating the effects of these turbulent forces through active control strategies is an ongoing challenge for both UAV and wind turbine applications^3–9^. However, turbulent conditions in the atmosphere are highly nonstationary and nonlinear making them difficult to model or control in real-time^1,10^.

Biological systems have long inspired engineers aspiring to develop systems robust to chaotic environments. Along with inertial and visual cues, sensed through vestibular, proprioceptive, or ocular systems, some animals navigate turbulent and unsteady environments through the use of biological flow sensors^11^. For example, the lateral line is a sensory system common to most fish species, and is typically made up of hundreds of neuromasts located all along the fish’s body^12^. The flow information observed by the lateral line allows fish to sense disturbances remotely, which can be used for finding prey, avoiding predators, achieving schooling formations, and navigating turbulent waters^13–16^. Similarly, often admired for their acrobatic flight capabilities, bats have a set of microscopic hairs located on their wings that sense airflow and may enable enhanced control of unsteady aerodynamics^17–19^. Finally, large sea birds of the Procilliforme family (e.g. Wandering Albatross, Giant Petrel) use their tubular-shaped nostrils to sense and ride turbulent air gusts from breaking waves, allowing for long-distance flight with minimal energy expenditure^20^.

The impressive capabilities of these biological systems have previously motivated research for enhanced control of underwater and aerial autonomous vehicles through bio-inspired flow sensors^21–28^. This work has generally implemented basic proportional-integral-derivative (PID) control techniques when testing flow sensory systems. While PID control is very effective for systems that can be linearized, the dynamics of turbulent flow are strongly nonlinear and cannot be reduced to even locally linearized approximations^10,29,30^. To properly realize the potential of bio-inspired flow sensing for control, nonlinear control methods are needed.

**Fig. 1:**
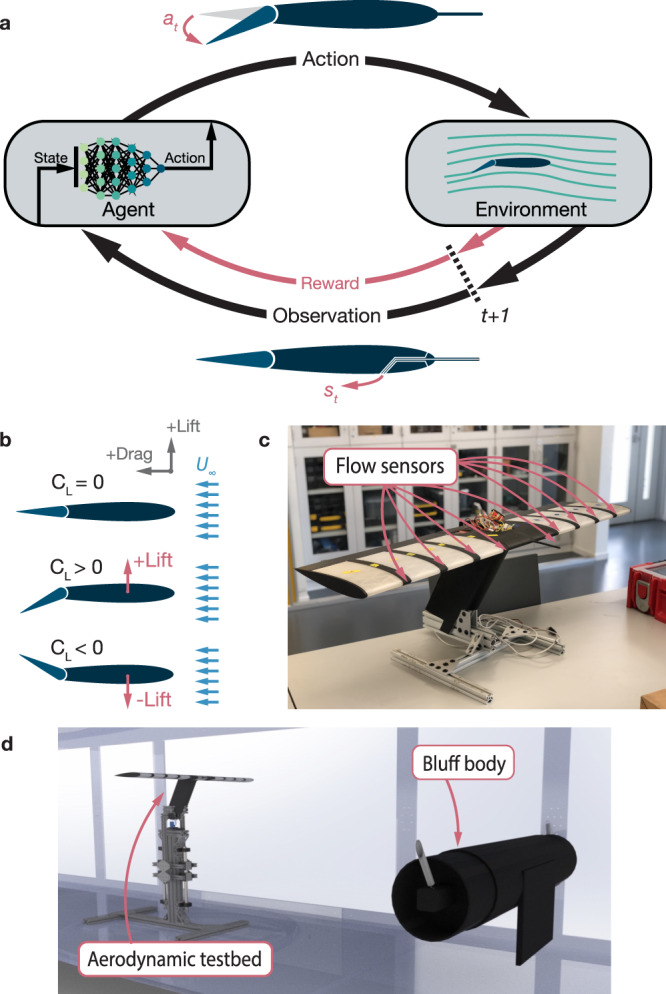
Basics of experimental setup and design. **a** Schematic depicting the use of a Markov decision process framework, where agents take actions based on their observations of their environment, to address the problem of turbulent disturbances. **b** Symmetric airfoils produce zero lift when aligned with uniform flow. Changing the position of a trailing edge flap can change the coefficient of lift and create an aerodynamic force either upward or downward. **c** The wing system used for training featured a modular design, as shown by the different color sections. Each section is removable and replaceable, and the locations of flow sensors are labelled. The wing is 1 m in length (see Methods for more details). **d** The system was trained in the wake of an asymmetric bluff body in a conventional closed-loop wind tunnel. The cylindrical portion of the bluff body has diameter of 30 cm, and was placed 170 cm upstream of the wing system.

Model-free reinforcement learning (RL) is a machine learning framework that can be formulated to control nonlinear systems without any prior knowledge or modeling of the system dynamics. RL methods were principally inspired by biological learning theories regarding how animals learn new behaviors through repeated trial-and-error^31^. In RL, these trial-and-error interactions fit the formal framework of a Markov decision process framework (Fig. 1a), where discrete time steps consist of observing the state of the environment and choosing an action based on that observation. A numerical reward associated with each previous interaction is used to learn and improve the action decision-making. Observations can be comprised of any available state information, which the agent learns to interpret through experience alone. Actions can consist of any actuations or manipulations that the agent can physically realize. Capable of learning control policies and observation interpretations through direct interactions with physical phenomena, it may hold potential for flow-informed aerodynamic control in turbulent, non-stationary conditions^32^.

Here, we experimentally investigate the use of state-of-the-art RL methods provided integrated flow information for aerodynamic control in a highly turbulent and vortical environment. RL algorithms are implemented on an aerodynamic testbed consisting of a wing with actuated trailing-edge flaps. Responding to incoming disturbances at each time-step by adjusting the position of the trailing-edge flaps to control the aerodynamics of the system, we set the goal of minimizing the standard deviation of lift in an unsteady, turbulent flow field. The wing system features an array of pressure sensors used to observe the aerodynamic state, and is mounted on a load-cell which can be used to observe the inertial state. We use recurrent neural networks (RNNs) to improve learning in a highly stochastic and partially observable physical environment that cannot be simulated trivially in a computational setting. Through these wind tunnel experiments, we show that RL agents are able to effectively integrate flow knowledge and achieve superior disturbance rejection relative to a conventional linear controller (i.e. PID). Overall, we find that model-free RL methods are capable of learning and controlling physical aerodynamic systems in turbulent and highly irregular flow fields.

## Results

### Flow-informed aerodynamic testbed

The problem of an aerodynamic system in turbulence was abstracted and generalized to a basic setting. We developed a testbed consisting of a symmetric airfoil with motorized trailing-edge flaps and integrated flow sensors. A generic platform such as this is ideal for aerodynamic study as it can easily be abstracted to more specific applications such as fixed-wing UAVs and wind turbine blades. In a uniform flow symmetric airfoils have lift coefficient ${C}_{L}=0$ at ${0}^{\circ }$ angle-of-attack, which means no aerodynamic force is produced in the upwards or downwards direction. As illustrated in Fig. 1b, the lift coefficient of a symmetric airfoil in a uniform flow can be manipulated by adjusting the position of a trailing-edge flap which results in a non-zero force along the lifting axis.

The wing system (Fig. 1c) was designed to be modular, allowing for variation of sensor type and placement. For this work, the model was configured to include nine sensors at different positions placed 10 cm apart from one another along the spanwise axis. The center position sensor featured a pitot-static tube for measuring mean flow velocity. The remaining eight sensors consisted of pressure taps placed at various chord lengths near the leading edge (details in Methods). The locations of the sensors were chosen based on previous works for aerodynamic parameter estimation from sparse on-body flow measurements^33,34^.

### Turbulent environment

**Fig. 2:**
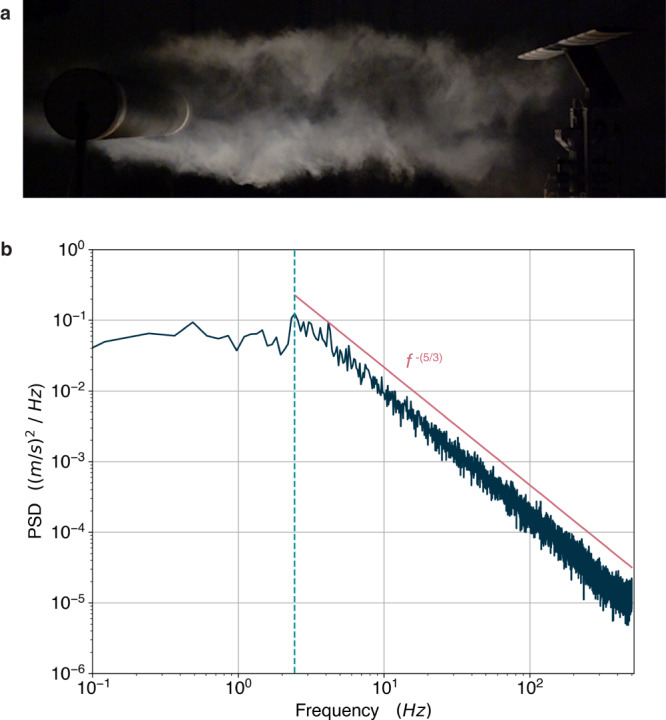
Characterization of turbulent flow conditions. **a** Smoke visualizations showing the turbulent wake of a standard cylinder at Reynolds number $R{e}_{D}\approx {{{{\mathrm{50,000}}}}}$. This was taken in the Caltech Center for Autonomous Systems and Technologies fan-array wind-tunnel at a lower Reynolds number for purposes of visualization. The actual flow conditions used for testing and training were too turbulent for effective smoke visualization. **b** Power spectrum measured in the bluff-body wake plotted logarithmically. We note the peak frequency at 2.44 Hz (dashed line), the relative width of the high-energy low-frequency region (left of dashed line), and the –5/3 slope energy decay that agrees with turbulence theory (right of dashed line)^39,40^.

Bluff body wakes are a well-studied problem in fluid mechanics. Famously, these wakes can feature a phenomenon commonly known as a Kármán vortex street which consists of alternating vortices shedding at a fixed frequency. However the behavior of vortex shedding in a bluff body wake can change, and there exists a well-established relationship between this behavior and the Reynolds number of the flow^35–38^. At a sufficiently high Reynolds number the wake becomes turbulent and vortex shedding, while still present, becomes less regular (Fig. 2a)^36^. To simulate a gusty environment, we placed our wing system in the wake of a large, asymmetric bluff body which was mounted on elastic bands in a wind tunnel (Fig. 1d). The asymmetry of the body and dynamic mounting produces highly irregular turbulent disturbances. This flow field is not intended to exactly match any specific atmospheric conditions, but rather to create challenging environment with frequent large amplitude vortical disturbances.

A hot-wire anemometer, placed near the leading edge of the wing, characterized the velocity, turbulence intensity, and frequency spectrum of the flow (see Methods for details). The mean velocity was recorded as 6.81 m s^−1^, which corresponds to a Reynolds number of approximately $R{e}_{D}\approx$ 230,000 over the bluff body. Figure 2b depicts the power spectral density calculated from the hot-wire anemometer measurements. Here we see a peak at 2.44 Hz, which can be assumed to be the primary vortex shedding frequency. This corresponds to a Strouhal number of ${St}=$ 0.19, which is in good agreement with the expected value for a bluff body wake at this Reynolds number^36^. However, the power spectrum also suggests that there is much energy stored at frequencies lower than the primary vortex shedding frequency, given the width of the high-energy, low frequency region. This indicates that flow disturbances are highly irregular in length and time scales and demonstrates the presence of gust-like disturbances arriving at random intervals. We also note the energy decay beginning at the primary shedding frequency which follows a −5/3 power law, agreeing with theory of the turbulent energy cascade^39,40^.

### Model-free reinforcement learning

To achieve flow-informed aerodynamic control of our wing system, we implemented the twin delayed deep deterministic policy gradient algorithm (TD3) as well as variant known as LSTM-TD3^41,42^. These are off-policy actor-critic type algorithms which use neural networks to make control policy decisions (see Methods). TD3 was previously deployed successfully for experimental flow control in a different setting^43^. LSTM-TD3 features a modification to the neural network structure of TD3 to include recurrent long-short-term-memory (LSTM) cells. The presence of recurrent cells in neural networks can considerably improve performance in partially observable systems, which can impact performance and prediction in highly stochastic settings such as the development of turbulent flows^42,44^. Since training RL algorithms is an inherently stochastic process, we trained each of the agents presented here with five separate random seedings and averaged the results to show general performance. Each agent was trained for 200 episodes which took approximately 150 min per agent.

Through training, RL algorithms attempt to learn control policies that maximize a numerical reward signal which is prescribed beforehand to set the desired goal of learning. We designed our reward to hold a constant lifting force (arbitrarily set to zero) in the presence of flow disturbances, setting it equal to ${R}_{i}=-{\left({L}_{i+1}\right)}^{2}$, where ${R}_{i}$ is the reward at timestep $i$, and ${L}_{i+1}$ is the lifting force at timestep $i+1$ (see Methods for details). A perfect system would achieve zero lifting force at each timestep, giving a maximum possible reward equal to zero. We can use the mean accumulated reward of each episode as a measure for comparison between algorithms and as a basic indicator of learning behavior. As the name implies, the mean accumulated award is the sum of rewards accumulated in a single episode averaged across the five agents. We also use the standard deviation of lift calculated from the time-histories of each episode to evaluate performance directly related to disturbance rejection.

### Comparison with baseline control methods

As a metric of baseline performance, we compared the RL algorithms with basic PID control. The PID controller was tuned manually and set to achieve constant zero lift with feedback from forces measured by the load cell. LSTM-TD3 and TD3 agents were provided near identical network parameters (see Methods). To gauge how the respective control policies reduce the effect of flow disturbances, we also measured the wing system sitting passively in the turbulent environment for comparison. Due to the randomized nature of training model-free algorithms, performance and consistency of learning are both important metrics when evaluating the behavior of RL agents. The fully trained RL agents, PID control, and passive configuration were set to perform over approximately one minute each, and the resulting standard deviations of lift and calculated episodic mean accumulated reward are shown in Table 1. PID offered only modest improvements in disturbance rejection over no control, with a ~13% reduction in standard deviation of lift. The TD3 algorithm had a similar reduction in standard deviation as PID, however also demonstrated large variation between agents, indicating inconsistency in its ability to learn the system dynamics. The LSTM-TD3 algorithm performed well, reducing standard deviation of lift by 42% relative to the passive case. Noting that the maximum reward possible is zero, we also see that TD3 accumulates a negative value over five-times greater than that of LSTM-TD3. Additionally, as indicated by the uncertainties, LSTM-TD3 was also more consistent across agents despite the stochasticity of training. These uncertainties, calculated as the standard deviation of the respective quantities across the five agents are themselves indicators of training stability.

**Table 1:** Statistical comparison of control schemes

Algorithm/controller	Standard deviation of lift (mN)	Mean accumulated reward
No control	$305\pm 20$	N/A
PID	$264\pm 6$	N/A
TD3	$266\pm 79$	$-8960\pm 10728$
LSTM-TD3	$176\pm 11$	$-1716\pm 452$

Rewards and standard deviation of lift values were averaged over five agents trained for both of the reinforcement learning algorithms and five separate runs for the proportional-integral-derivative (PID) control. They were calculated over a 4000 time step horizon, which corresponds to approximately 1 minute of testing or four-times the length of a training episode. Uncertainty shown is equal to the standard deviation in the presented value across five separate training sessions. Supplementary figure 1 shows examples of the load signal over a 60 s interval for all four methods listed in the table below.

**Fig. 3:**
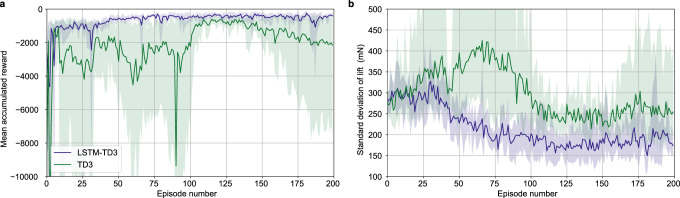
Training performance of TD3 and LSTM-TD3. The respective shaded regions represent the full range of performance of each algorithm at each episode. **a** Learning curve showing the episodic mean accumulated reward across the five agents trained for each algorithm. **b** Episodic standard deviation for the two algorithms as it decreases with training.

Further, the mean accumulated reward (per episode) plot (Fig. 3a) indicates that LSTM-TD3 agents consistently improved throughout training, whereas the TD3 agents struggled to find even locally optimal behaviors. While the reward signal returned for the TD3 agents became less erratic with training, it eventually decays and showed a downward trend during the final episodes with increased variance. This suggests that the dynamics learned by TD3 are not representative of the real system. As expected from the uncertainty in the standard deviation of lift (Table 1), we can confirm that the learning process for the TD3 is inconsistent and there exists variation across the separately trained agents. The episodic standard deviation of lift (Fig. 3b) remains relatively stable after approximately 100 episodes of training for both algorithms. The lack of reduction in standard deviation of lift from both agents across such a large span of episodes suggests that they have reached asymptotic performance for the given conditions. Despite these two methods being nearly identical algorithmically, it seems that the simple inclusion of RNNs in LSTM-TD3 makes aerodynamic control tractable in this turbulent environment.

### Effect of flow sensing

Conventional control strategies for UAVs mitigate turbulent disturbances by sensing and correcting the resulting inertial deviations. They have no knowledge of the flow or source disturbance itself. This purely reactive-corrective strategy is insufficient for maintaining stability under extreme atmospheric turbulence^45^. Alternatively, as biological swimmers and flyers would imply, directly observing the physics responsible for inertial disturbances may allow for aerodynamic systems to react before inertial effects are realized. The flow sensing capabilities of biological systems can then be used as inspiration to improve these strategies, given the direct correlations between easily measurable flow quantities, such as pressure, and aerodynamic forces.

To show the effect of flow sensing on the performance of aerodynamic control in turbulence, we conducted ablation studies wherein we maintained the same reward signal but varied the sensory information provided to the agent. We considered three cases to establish how flow sensing impacts the ability to learn system dynamics. In Case I, the RL agents observed and chose actions based on the value of the lift force alone through the real-time load cell values. Observing only the lift force, the RL agent in Case I were effectively provided with inertial information equivalent to conventional UAV controllers. In Case II, actions were selected using only flow measurements as the observation, though the lift measurements were used to calculate rewards during an offline training phase. In Case III, the agents observed both the lift force and flow measurements. With both inertial and flow information, Case III was afforded a set of information similar to a flow-sensing biological flyer. All three cases were trained using the LSTM-TD3 algorithm; they differed only in the sensory information provided to the agent for action selection.

**Fig. 4:**
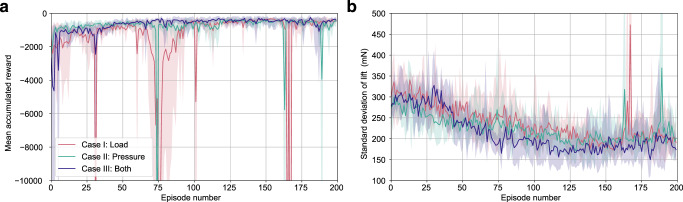
Training performance of reinforcement learning algorithms with varying observations. The respective shaded regions represent best and worst performing agent of each case at each episode. **a** The learning curves for the three respective cases, plotting episodic mean accumulated reward. **b** The change in standard deviation throughout learning plotted as a metric for disturbance rejection.

From a comparison of the respective episodic reward signals (Fig. 4a), we found that the three cases seem to learn similarly effective control strategies in after training 200 episodes. While all three cases occasionally experienced policy updates that decreased performance (as indicated by downward spikes), these detrimental updates appeared most frequently and most strongly for the Case I agents. The Case II agents also had several notable bad policy updates, but recovered more quickly than the Case I agents. The Case III agents learned more stably and reliably than the Case I and Case II agents, with the best Case III agents consistently outperforming the best Case I and Case II agents throughout training, and with the worst Case III agents rarely performing worse than equivalent Case I and Case II agents (highlighted regions-Fig. 4a). Case III agents also achieved the lowest mean standard deviation of lift and lowest minimum standard deviation across agents for most episodes throughout training (Fig. 4b).

In addition to considering the performance during learning, we also compared performance of the fully trained RL agents for all three cases. We averaged the performance of fully trained models for all three cases over a time interval of approximately one-minute to reduce the effect of stochasticity in the flow. From the final time-averaged standard deviation of lift values for the three cases (Table 2) we see all three agents considerably reduced the variance of lift relative to the passive case. We find that that Case III shows superior performance in reducing the standard deviation of lift and is the most consistent in that metric across the five separately trained agents. Both the training process (Fig. 4) and the final standard deviation (Table 2) suggests that the addition of flow sensing helps RL agents learn a more stable approximation of the system dynamics and improved performance in terms of disturbance rejection (i.e. reduction of standard deviation).

**Table 2:** Statistical comparison of lift force with and without flow sensing

Case	Standard deviation of lift (mN)	Mean accumulated reward
No control	$305\pm 20$	$\rm N/A$
I (Load)	$191\pm 20$	$-1696\pm 492$
II (Pressure)	$199\pm 33$	$-1860\pm 596$
III (Both)	$176\pm 11$	$-1716\pm 452$

These values were averaged over five agents trained for each of the respective cases, and were calculated over a 4000 time step horizon, which corresponds to approximately 1 minute of testing or four-times the length of a training episode. Uncertainty shown is equal to the standard deviation in the presented value across five separate training sessions. Supplementary Fig. 2 shows examples of the load signal over a 60 s interval for the three different observations.

Interestingly, we find that Case I achieves the best (least negative) mean accumulated reward out of the three cases, with Case III falling closely behind by only a small margin. We note that the uncertainty reported for the reward of these two cases is approximately twenty-times the apparent difference in performance, which reduces the significance of this comparison. Still, it is not surprising that the Case I performance excels in terms of raw reward, as the only observation given in Case I is directly proportional to the reward itself. Considering that the Case I agents achieve a higher standard deviation of lift, the performance in terms of reward is the result of the agents holding a lower mean lift. Although Case I agents are given less information about the surrounding physics, the information they are given has only one dimension and excludes highly non-linear flow sensor readings. These attributes would enable a more simple and less sensitive control policy which simultaneously explains a lower mean lift value and less responsive disturbance rejection.

## Discussion

We have demonstrated how properly configured RL agents can effectively learn control of nonlinear stochastic physics with which conventional methods struggle. Despite the seemingly chaotic nature of the turbulent environment used for training, our results indicate that RNNs enhance the ability to learn accurate system dynamics. Further, the inclusion of flow sensors, as inspired by biological systems, showed potential for enhanced aerodynamic control in turbulence.

We found that the performance achieved by the TD3 agents was very similar to that of a conventional PID controller (Table 1). This result was surprising, as the TD3 agent should be able to better handle the non-linearities of the system dynamics than the inherently linear PID controller. The poor performance of the TD3 algorithm (in comparison with LSTM-TD3) may be explained by the partial observability of our system. It is likely that observing inertial and sparse flow measurements at a single time-step does not adequately define the state; the probability distribution of state transitions is dependent on the surrounding flow which is chaotic and impossible to fully observe in real. Therefore, without being explicitly given a more comprehensive state observation, the TD3 agents are unable to infer the underlying state and effectively learn the underlying physics. This difficulty to learn the underlying physics of the problem would help explain the large variation in both training and end performance for the TD3 agents (Fig. 3).

We showed that LSTM-TD3 agents were able to achieve effective control of the system aerodynamics by analyzing the available observations sequentially, and outperformed both PID controllers and TD3 agents. In fact, the LSTM-TD3 agents were able to decrease the standard deviation in lift by more than three-times the reduction achieved by PID control. Further, despite being trained in five separate randomized processes, the final performance of the LSTM-TD3 agents is nearly as consistent as the five trials used to average the fixed PID controller. This demonstrates the ability to learn accurate estimations of the state-probability distribution functions. Due to the similarity of the two algorithms, the addition of recurrent LSTM cells is very likely the reason for the difference in performance between the TD3 and LSTM-TD3 agents. The potential performance-enhancing nature of LSTMs is well-established in many settings, including flow-control^42,46^. When included in an RL agent, recurrent networks, such as those including LSTM cells, are able to learn latent states and patterns underlying the received observations. Because of the black-box nature of these methods, it is only possible to speculate what aspects of the physics were learned through the addition of LSTM cells. However, the fundamental mechanism through which LSTM cells can typically improve performance is by integrating temporal information to improve state estimates. Therefore, the increase in performance associated with the LSTM cells is likely due to improved state estimates which would effectively increase the observability of the partially-observable process.

Flow sensing was shown to slightly improve mitigation of turbulent disturbances for our system, although it resulted in a larger bias in the averaged value than inertial information alone. Still, when provided flow and inertial information the RL agents did learn more consistently and achieved superior disturbance rejection than when given partial information (Fig. 4). Further, the fully trained control policies given both flow and inertial information varied less in all metrics, suggesting more stable estimates of system dynamics. It is also noteworthy that agents observing only flow measurements showed robust control improvement through learning; that is, agents were able to learn to control inertial dynamics from sparse flow sensing alone. While the load cell used for testing completely defines the inertial state of the system in the lifting direction, the pressure sensors used to make flow observations are relatively sparse and do not completely define the aerodynamic state in the lifting direction. It is likely that the performance of flow-informed agents would increase further given additional or improved flow sensing capabilities, while the inertial aspects of the lifting force cannot be defined further than the direct measurement used here. This warrants further exploration of flow sensor types and configurations.

Though the RL controllers outperformed a conventional linear control scheme, there are several drawbacks to model-free reinforcement learning methods. Training RL agents is intensive in terms of both time and data. Each RL agent was trained for 200 episodes which took approximately 150 min per agent. Since we averaged the performance of five agents for each algorithm or case shown, the data presented here represents over 50 wind-tunnel hours between training and testing the policies. There are also inherent difficulties associated with troubleshooting “black-box” controllers such as RL agents. The algorithms are sensitive to many hyperparameters that control the neural network structure and training procedure, and tuning these hyperparameters in an experimental setting relies on intuition, experience, and patience. The hyperparameter tuning process itself required hundreds of hours of additional training and testing not shown here. Further, it is possible that a given set of hyperparameters may be suitable for a subset of tasks but not truly generalizable. Consistent and deliberate experimental design helps constrain troubleshooting to the algorithmic aspects of training. Even with this, it should not be expected that these agents trained in a single set of conditions will hold policies generalizable across Reynolds numbers or testing geometries. To create truly generalizable RL policy capable of controlling an aerodynamic system ready for real-world deployment, the agent would need to train in various conditions and would need to expand its capabilities to control all forces and moments in three-dimensions.

While model-free reinforcement learning methods impressively learn dynamics of highly nonlinear and chaotic systems without any prior knowledge, it should be noted that model-based reinforcement learning and other non-linear control methods can be more data-efficient. Model-based methods do require prior sampling of system dynamics and can be more computationally intensive, but many are similarly able to adapt and learn when exposed to new conditions. Implementing known flow physics into model-based reinforcement learning methods or non-linear controllers could lead to superior performance with reduced data requirements.

The generic testbed and methods developed here may serve to inform future implementations of flow-informed RL for control of aerodynamic systems in extreme turbulence. While our experiments focus on specifically controlling turbulent disturbances along a single axis, this system is simply a representative proof-of-concept for multi-dimensional unconstrained aerodynamic interactions. These methods can be expanded or adapted to systems with higher degrees of freedom by augmenting state observations and adjusting reward signals. While it is possible for RL methods to be used for full control and navigation of autonomous systems^47,48^, the most direct and practical application of aerodynamic control for UAVs is in flow-informed inner-loop attitude control for fixed wing vehicles. By reducing the effect of turbulent disturbances, drones can maintain more stable flight in more extreme conditions. Though training RL agents to achieve full control of free-flight systems can be challenging experimentally due to the trial-and-error nature of the learning process, flow-informed agents even have the potential to learn to take advantage of natural flow structures through energy-efficient soaring behaviors^20,47,49^. This technology could also allow wind turbines to safely operate at an increased range of conditions by reducing loads from potentially damaging gusts through actuation of blade pitch^3,5,7^. In the case of static systems such as wind turbines, off-board remote sensing upwind could further enhance performance. We believe that the potential of this work can be realized through several next generation technologies such as flow-informed wind turbines with built-in gust mitigation capabilities, bioinspired UAVs capable of maintaining steady flight in a windy urban environment, and other unrealized aerodynamic applications that have been too chaotic for engineered systems.

## Methods

### Wing system design and manufacturing

The wing system featured a NACA0012 airfoil, which is a common standardized airfoil shape. The dynamics of this airfoil shape in a bluff-body wake at similar Reynolds number has been the subject of previous study^50^. The body of the wing was 3D printed using a combination of materials, and was designed to be modular and allow for various sensor configurations (Fig. 1c). The central section, which housed the primary electronics and secured the system to its mounting, was printed with micro carbon fiber filled nylon (Markforged Onyx) and was reinforced with carbon fiber for added strength and rigidity. The spanwise sections designed to house the individual pressure sensors were also printed with micro carbon filled nylon, but were not reinforced. The sensor housing sections were printed with large slots, so that different pressure taps or probe types could be used. These pressure tap slots and the pitot-static tube were printed with an SLA printer (Formlabs Form3) for improved surface feature accuracy. The pressure ports were placed at locations 0.4%, 0.7%, 1.5%, and 6% of the chord length from the leading edge on both the pressure and suction sides of the wing. The sections between sensors were printed with clear PLA. The sections were aligned and conjoined by a set carbon fiber spars, which added rigidity. The trailing edge flaps were cut out of insulation foam and covered with an adhesive-backed coating for protection.

The wing had a total chord length of 25 cm, with 5 cm trailing edge flaps. This gave a Reynolds number over the wing of approximately $R{e}_{c}\approx {{{{\mathrm{110,000}}}}}$. The spanwise length of the wing was 1 m, with a total of 9 sensor locations. There was exactly 10 cm between each sensor location, with one of the locations being centered on the wing. The wing was mounted on a fairing which was set back with an angle of 60° to reduce aerodynamic interactions between the fairing and the wing. The fairing was reinforced with carbon fiber and aluminum, and was connected to a set of air bearings (New Way) which are aligned vertically with the tunnel to define the lifting direction. The air bearing system allowed for nearly frictionless motion along a single axis while constraining all other directions. The constrained fairing was mounted directly to a single-axis load cell (Interface SM-50), which passed signal through an amplifier (Interface Model SGA) with a 50 Hz low pass filter, and was read by a DAQ (NI USB-6008). The pressure values were measured by a set of nine ultra-low range digital pressure sensors (Honeywell RSCDRRM2.5MDSE3) which communicated to a microcontroller (Teensy 4.0). The microcontroller also controlled the high speed brushless servo motors (MKS HBL6625) which drove the trailing edge flaps. Due to mechanical restraints, the actuation for the servo motors has maximum/minimum position of +40°/−40°. Both the microcontroller and the DAQ communicate with a desktop computer which receives states and sends actions. The full control loop ran at approximately 67 Hz with the serial communications being the biggest bottleneck.

### Generation and characterizations of turbulence

All quantitative results presented are from experiments performed in Caltech’s John W. Lucas Wind Tunnel (LWT). The LWT is a closed-loop wind tunnel, with test section dimensions of 130 cm $\times$ 180 cm. The turbulence used for training and testing formed in the wake of a large, asymmetric bluff body mounted to the wind tunnel with bungee cords. The bluff body can be described as a large diameter cylinder (30 cm), with a normal flat plate mounted asymmetrically to the front giving the full body an effective diameter of 53 cm (Fig. 1d). The cylinder spanned the entire width of the tunnel, while the flat plate had a width of only 60 cm. This was done to encourage vortex dislocation, which added irregularity in vortex shedding^38^. The bungee cord encouraged oscillations due to the vortex shedding, which we observed to be present and irregular. The bluff body was mounted 170 cm upstream of the wing system with a vertical offset of 48 cm. Sparse elastic bands aligned horizontally were mounted across the test section directly upstream of the bluff body, to further increase the turbulence intensity of the flow.

A hot-wire anemometer (TSI IFA-300) was used to characterize the mean velocity, turbulence intensity, and frequency spectrum of the flow near the wing system. The hot-wire anemometer was mounted approximately 2 cm upstream of the leading edge of the airfoil, and measurements were taken for 120 s at 1000 Hz. The turbulence intensity was measured with the hot-wire anemometer to be 10.6%. The power spectra was calculated with ThermalPro software, using the entire 120 s run averaged over sets of 8192 datapoints (frequency resolution of 0.122 Hz).

### Reward functions for training

Reinforcement learning agents learn to achieve tasks by choosing actions that maximize a numerical reward function. Choosing the reward function is a critical part of experimental design for RL implementations, as it sets the primary directive of learning. The goal of our experiments was to reduce the standard deviation of the lifting force, and we tested several different reward functions to achieve this goal. The final reward function used in this work was set to

$${R}_{i}=-{\left({L}_{i+1}\right)}^{2}$$

where ${R}_{i}$ is the reward associated with the action taken at time step $i$, and ${L}_{i+1}$ is the lifting force observed at the time step $i+1$. The reward was a function of the lift at the following time step rather than the current time step, because this value is the direct result of the previous action. Since RL agents are designed to maximize reward signals, we used the negative square of this value to encourage net-zero lifting force and discourage deviations. Under these conditions, a perfect agent would achieve a maximum reward equal to zero. Although this reward function may seem overly simplistic, it was chosen out of several tested all of which featured additional terms that did not improve performance. We note that this reward function was even used for the agents trained with flow sensor values only. The agents in this case had no direct knowledge of the lift while making decisions, and it was measured and saved separately from the state observations. These flow-only agents were only affected by the actual lift measurements through off-line training with the reward as shown.

### Reinforcement learning algorithms

Both the TD3 and LSTM-TD3 fall in a category of RL algorithms known as actor critic methods. As the name suggests actor critic methods consist of two parts, the actor and the critic. The actor portion holds the direct control policy of the agent; it is called at each time step, with observations as inputs and actions as outputs. The critic portion is used to estimate the value of each available action given a state. The value of an action is equal to the expected cumulative future reward^31^. In most modern applications, both actors and critics take the form of artificial neural networks. The critic networks become approximators for the value function, and the actor network becomes an approximator for an optimal control policy. The agent learns by first training the value function estimation of the critic based on past state-action-reward experiences. The actor is then trained using the critic network to choose the actions which the critic estimates will have the highest expected value. Actor critic methods are known to have reduced variance in updates, which accelerates learning and makes them well-suited for real-world applications^31^. The TD3 (provided in Algorithm 1) and LSTM-TD3 (provided in Algorithm 2) algorithms build on this basic framework with several modifications to improve performance.

#### Algorithm 1

TD3 algorithm used for aerodynamic control^41,43^.

Initialize critic networks ${Q}_{{\theta }_{1}},\,{Q}_{{\theta }_{2}}$, and actor network ${\pi }_{\phi }$ with random parameters ${\theta }_{1},\,{\theta }_{2},\,\phi$

Initialize target networks ${\theta }_{1}^{{\prime} }\leftarrow {\theta }_{1},\,{\theta }_{2}^{{\prime} }\leftarrow {\theta }_{2},\,\phi^{{\prime}} \leftarrow \phi$

Initialize replay buffer ${{{{{\mathcal{B}}}}}}$

**for** $n=1$ **to** ${N}_{e}$ **do**

 **for** $t=1$ **to** $T$ **do**

  Observe state $${o}_{t}$$

  Select action with exploration noise:

  $${a}_{t}\leftarrow {{{{{\rm{clip}}}}}}({\pi }_{\phi }({o}_{t})+{\epsilon }_{t},-{{{{\mathrm{1,1}}}}}),\,{\epsilon }_{t}{{{{{\mathscr{ \sim }}}}}}{{{{{\mathscr{N}}}}}}(0,{\sigma }^{2})$$

  Observe reward $${r}_{t}$$

 **end for**

 Store transition tuples ${\left\{\left({s}_{t},{a}_{t},{r}_{t},\,{s}_{t+1}\right)\right\}}_{i=1}^{T-1}$ in ${{{{{\mathcal{B}}}}}}$

 **for** $j=1$ **to** ${N}_{s}$ **do**

  Sample $N$ transitions $(s,a,r,{s}^{{\prime} })$ from ${{{{{\mathcal{B}}}}}}$

  $$\widetilde{a}\leftarrow {{{{{\rm{clip}}}}}} ({\pi }_{{\phi }^{{\prime} }}({s}^{{\prime} })+\epsilon ,\,-1,\,1),\,\epsilon \sim {{{{{\rm{clip}}}}}} ({{{{{\mathscr{N}}}}}}(0,{\widetilde{\sigma }}^{2}),\,-c,{c})$$

  $$y\leftarrow r+\gamma \,{{{\min }}}_{i={{{{\mathrm{1,2}}}}}}\,{Q}_{{\theta }^{{\prime} }}\left({s}^{{\prime} },\,\widetilde{a}\right)$$

  Update critics $${\theta }_{i}\leftarrow {{{\min }}}_{{\theta }_{i}}{N}^{-1}\sum \left\{\begin{array}{c}{(y-{Q}_{{\theta }_{i}}(s,a))}^{2},{|y}-{Q}_{{\theta }_{i}}(s,a)|\le \delta \\ \delta * (|y-{Q}_{{\theta }_{i}}\left(s,a\right)|-\frac{1}{2}\delta ),{{{{{\rm{else}}}}}}\end{array}\right.$$

  **if** $j$ mod $d$ **then**

   Update *ϕ* by the deterministic policy gradient:

   $${\nabla }_{\phi }J\left(\phi \right)={N}^{-1}\sum {\nabla }_{a}{Q}_{{\theta }_{1}}\left(s,a\right){|}_{a={\pi }_{\phi }\left(s\right)}{\nabla }_{\phi }{\pi }_{\phi }(s)$$

   Update target networks

   $${\theta }_{i}^{{\prime} }\leftarrow \tau {\theta }_{i}+\left(1-\tau \right){\theta }_{i}^{{\prime} }$$

   $${\phi }_{i}^{{\prime} }\leftarrow \tau {\phi }_{i}+\left(1-\tau \right){\phi }_{i}^{{\prime} }$$

  **end if**

 **end for**

**end for**

#### Algorithm 2

LSTM-TD3 algorithm used for aerodynamic control^42^.

Initialize critic networks ${Q}_{{\theta }_{1}},\,{Q}_{{\theta }_{2}}$, and actor network ${\pi }_{\phi }$ with random parameters ${\theta }_{1},\,{\theta }_{2},\,\phi$

Initialize target networks ${\theta }_{1}^{{\prime} }\leftarrow {\theta }_{1},\,{\theta }_{2}^{{\prime} }\leftarrow {\theta }_{2},\,{\phi }^{{\prime} }\leftarrow \phi$

Initialize replay buffer ${{{{{\mathcal{B}}}}}}$

**for** $n=1$ **to** ${N}_{e}$ **do**

  Initialize past history ${h}_{1}^{l}\leftarrow 0$

  **for** $t=1$ **to** $T$ **do**

   Observe state $${s}_{t}$$

   Select action with exploration noise:

   $${a}_{t}\leftarrow {{{{{\rm{clip}}}}}}({\pi }_{\phi }\left({s}_{t},\,{h}_{t}^{l}\right)+{\epsilon }_{t},-{{{{\mathrm{1,1}}}}}),\,{\epsilon }_{t}{{{{{\mathscr{ \sim }}}}}}{{{{{\mathscr{N}}}}}}(0,{\sigma }^{2})$$

      Update history:

      $${h}_{t+1}^{l}=\left({h}_{t}^{l}-({s}_{t-l},{a}_{t-l})\right)\cup ({s}_{t},{a}_{t})$$

   Observe reward $${r}_{t}$$

 **end for**

 Store transition tuples ${\left\{\left({{h}_{t}^{l},s}_{t},{a}_{t},{r}_{t},\,{s}_{t+1}\right)\right\}}_{i=1}^{T-1}$ in ${{{{{\mathcal{B}}}}}}$

**for** $j=1$ **to** ${N}_{s}$ **do**

   Sample $N$ transitions $({s}_{t},{a}_{t},{r}_{t},{s}_{t+1})$ from ${{{{{\mathcal{B}}}}}}$

   $$\widetilde{a}\leftarrow {{{{{\rm{clip}}}}}}\left({\pi }_{{\phi }^{{\prime} }}\left({s}_{t+1},\,{h}_{t+1}^{l}\right)+\epsilon ,\,-1,\,1\right),\,\epsilon \sim {{{{{\rm{clip}}}}}}\left({{{{{\mathscr{N}}}}}}\left(0,{\widetilde{\sigma }}^{2}\right),\,-c,{c}\right)$$

   $$y\leftarrow {r}_{t}+\gamma \,{{{\min }}}_{i={{{{\mathrm{1,2}}}}}}\,{Q}_{{\theta }^{{\prime} }}\left({s}_{t+1},\,\widetilde{a},{h}_{t+1}^{l}\right)$$

   Update critics $${\theta }_{i}\leftarrow {{{\min }}}_{{\theta }_{i}}\,{N}^{-1}\sum \left\{\begin{array}{c}{\left(y-{Q}_{{\theta }_{i}}\left({s}_{t},{a}_{t},{h}_{t}^{l}\right)\right)}^{2},\,\big|y-{Q}_{{\theta }_{i}}({s}_{t},{a}_{t},{h}_{t}^{l})\big|\le \delta \\ \delta * \left(\big|y-{Q}_{{\theta }_{i}}\left({s}_{t},{a}_{t},{h}_{t}^{l}\right)\big|-\frac{1}{2}\delta \right),\,{{{{{\rm{else}}}}}}\end{array}\right.$$

   **if** $j$ mod $d$ **then**

      Update $\phi$ by the deterministic policy gradient:

      $${\nabla }_{\phi }{J}\left(\phi \right)={N}^{-1}\sum {\nabla }_{a}{Q}_{{\theta }_{1}}\left({s}_{t},{a}_{t},{h}_{t}^{l}\right){|}_{a={\pi }_{\phi }\left(s\right)}{\nabla }_{\phi }{\pi }_{\phi }({s}_{t},{h}_{t}^{l})$$

      Update target networks

      $${\theta }_{i}^{{\prime} }\leftarrow \tau {\theta }_{i}+\left(1-\tau \right){\theta }_{i}^{{\prime} }$$

      $${\phi }_{i}^{{\prime} }\leftarrow \tau {\phi }_{i}+\left(1-\tau \right){\phi }_{i}^{{\prime} }$$

   **end if**

  **end for**

 **end for**

We chose the original TD3 algorithm to start our tests because it is known to among state-of-the-art methods in RL and has been shown to outperform earlier methods in simulated tasks^51^. Further, the original TD3 algorithm was used previously effectively learn control in the first experimental application of RL for explicit control of fluid dynamics^43^. The LSTM-TD3 algorithm was chosen as a direct successor to the TD3 algorithm, which explicitly addresses the problem of partially observable systems^42^. Gradient clipping was added to both algorithms, to limit the size of updates and encourage training stability. Additionally, as suggested by a previous implementation of RL for experimental fluid mechanics, a Kalman filter was applied to both load and pressure data which was shown to considerably improve learning^43^. The hyperparameters used for both TD3 and LSTM-TD3 can be found in Table 3. We used densely-connected layers for both algorithms, with ReLU activation functions on the hidden layers and hyperbolic tangent for the output. The TD3 algorithm networks had just one hidden layer, and LSTM-TD3 had a separate input layer before the LSTM, then just one hidden layer after the concatenation of the LSTM output and current feature input.

**Table 3:** Hyperparameters used for training reinforcement learning algorithms

Hyperparameter	TD3	LSTM-TD3
Discount factor – $\gamma$	0.99	
Batch size - $N$	50	
Replay buffer size	50,000	
Target update rate – $\tau$	0.005	
Actor learning rate	${10}^{-3}$	
Critic learning rate	${10}^{-3}$	
Exploration noise - $\sigma$	0.025	
Policy smoothing noise - $\widetilde{\sigma }$	0.025	
Policy update delay - $d$	3	
Target noise clip boundary - $c$	0.5	
Actor gradient clip boundary	0.5	
Critic gradient clip boundary	0.5	
Optimizer	AMSGrad	
Timesteps per episode - $T$	1000	
Episodes trained ${N}_{e}$	200	
LSTM length $l$	N/A	10

These parameters were selected after manually tuning for both algorithms. We found that with similar network and algorithmic structures (the only difference being an LSTM branch for LSTM-TD3), the two methods performed best with the same parameters.

We chose hyperparameters based on metrics of peak performance and training stability. We used a methodical approach when selecting values however the search was necessarily coarse due to the time intensive nature of training. A more fine parameter search was not practical for our setting as each one of these tests took three hours, and there are many hyperparameters to consider. It was similarly impractical to perform repeated tests for most hyperparameters. Given these limitations, choosing parameter values required some subjective interpretation of agents’ performance, especially when several values appeared to perform similarly well.

The algorithms were trained episodically. Each episode began with a policy evaluation phase. During this phase, a fixed control policy was used to choose actions based on observations for a set number of time steps. This data was then saved for later evaluation of training. After the evaluation phase, the data collection period begins, which consisted of the agent with the same fixed control policy interacting with the environment for a set number of time steps, however, Gaussian noise is injected into the actions chosen by the policy to encourage exploration. Once the set number of time steps had been reached, all interactions and rewards from the data collection period are inserted into a replay buffer. Then the agent pauses interaction to train its neural networks. The critic network is trained by recalling interactions from a replay buffer that contains previous interactions from about 50 episodes of training. The actor network is then updated to maximize the value of actions based on the critic network value estimates. This policy evaluation, data collection, and training process completed a single episode. We chose to stop training after 200 episodes because we found that the LSTM-TD3 algorithm approached optimal performance around episode 100 but wanted to show a longer horizon to demonstrate the stability advantages of the algorithm.

### Supplementary information

Supplementary Material

## Data Availability

All raw experimental data is available on GitHub, along with an open-source access guide. Additional data may be available on request. https://github.com/peterirenn/ExpectingTurbulence.git.

## Code availability

All code used for this work is available on GitHub and is supported by open-source Python libraries. https://github.com/peterirenn/ExpectingTurbulence.git.
