# Supplementary material for: Machine learning for flow-informed aerodynamic control in turbulent wind conditions
Source: Commun Eng. 2022 Dec 16;1:45. doi: 10.1038/s44172-022-00046-z (PMC10955839; doi:10.1038/s44172-022-00046-z)
Supplement: Supplementary file 1 — Supplementary Material [file 44172_2022_46_MOESM1_ESM.pdf]

Supplementary Information

Expecting turbulence: machine learning for flow-informed aerodynamic control

Peter I. Renn1*, Morteza Gharib1

1Graduate Aerospace Laboratories, California Institute of Technology, Pasadena, CA 91125, USA
*Corresponding author. Email: prenn@caltech.edu

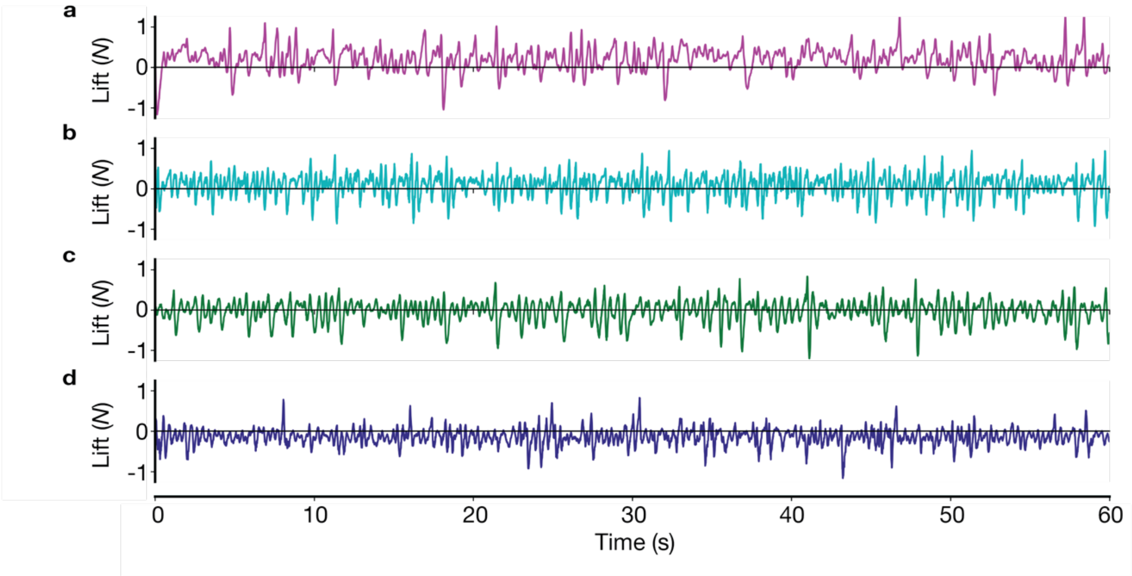

Supplementary Figure 1 Lift signal for various control methods. Randomly selectd examples of the lift signal over a 60 second interval for the control methods tested. (**a**) No control case. (**b**) PID control. (**c**) TD3 (**d**) LSTM-TD3.

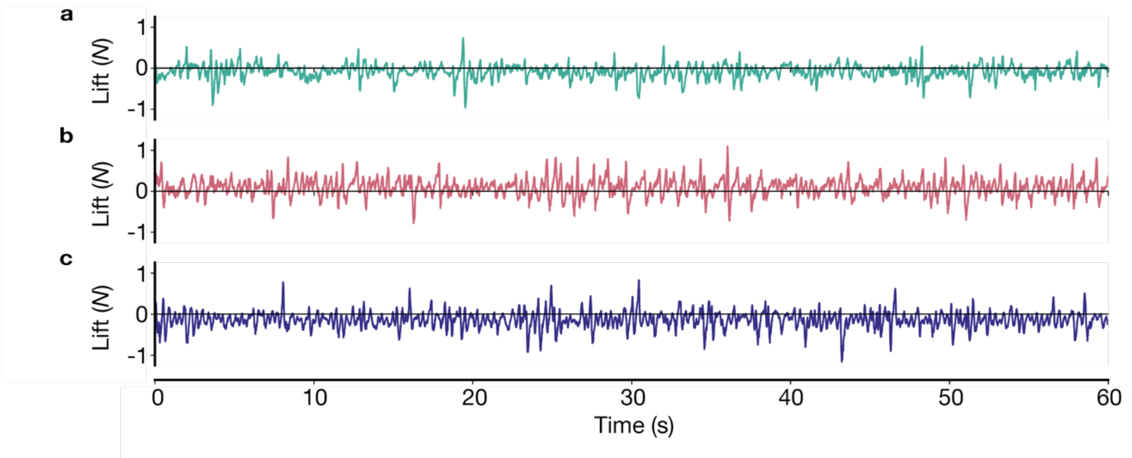

Supplementary Figure 2 Lift signal for various observations. The lift signal over a 60 second interval for the different observations tested. (**a**) Pressure only. (**b**) Load only. (**c**) Full observation.
